# Graph-based pangenomics reveals the genetic basis of agronomic traits in tomato fruits

**DOI:** 10.1093/hr/uhag147

**Published:** 2026-04-16

**Authors:** Xiao Su, Ran Du, Zijing Xing, Qinqin Yang, Yun Liu, Yan Wang, Yingfang Zhu, Jie Ye, Tao Lin

**Affiliations:** College of Horticulture, China Agricultural University, Beijing 100193, China; Key Laboratory of Synthetic Biology, Ministry of Agriculture and Rural Affairs, Agricultural Genomics Institute at Shenzhen, Chinese Academy of Agricultural Sciences, Shenzhen 518120, China; National Key Laboratory for Germplasm Innovation and Utilization of Horticultural Crops, College of Horticulture and Forestry Sciences, Huazhong Agricultural University, Wuhan 430070, China; College of Horticulture, China Agricultural University, Beijing 100193, China; College of Horticulture, China Agricultural University, Beijing 100193, China; College of Horticulture, China Agricultural University, Beijing 100193, China; State Key Laboratory of Crop Stress Adaptation and Improvement, School of Life Sciences, Henan University, Kaifeng 475001, China; National Key Laboratory for Germplasm Innovation and Utilization of Horticultural Crops, College of Horticulture and Forestry Sciences, Huazhong Agricultural University, Wuhan 430070, China; College of Horticulture, China Agricultural University, Beijing 100193, China

## Abstract

Tomato serves as a globally important vegetable crop and a genetic model organism, and the improvement of its agronomic traits represents a central objective in breeding. While graph-based pangenomes comprehensively capture species-wide genetic diversity, those built from large cohorts of individuals frequently present considerable computational challenges. To address this, we constructed a graph-based pangenome that integrated only the spectrum of genetic variants between wild (*Solanum pimpinellifolium*) and cultivated tomato (*Solanum lycopersicum*). By balancing reduced resource demands with reliable variant calling accuracy, this resource establishes a practical foundation for high-throughput genetic analysis. Using the graph-based pangenome and an F_7_ recombinant inbred line (RIL) population, genome-wide association studies (GWAS) pinpointed known loci for trichome, vegetative form, and weight traits, as well as a novel fruit-weight locus, *fw6.4*. The candidate gene *SlILL6* within this region likely affects fruit weight (FW) by modulating cell expansion. Metabolite profiling revealed micro-scale quality variation and identified 128 differentially accumulated metabolites. Among these, a GDSL lipase-like caffeoyltransferase (*SlCGT*) was found to function as a negative regulator of chlorogenic acid content, thereby affecting fruit resistance to pathogenic fungi. Integrating a graph-based pangenome with multi-omics data, this research deciphered the genetic basis of complex traits and supplies new genomic resources, analytical tools, and candidate genes for tomato improvement.

## Introduction

The tomato (*Solanum lycopersicum*) is one of the most valuable vegetable crops globally, serving as a key nutritional source for the human diet that greatly contributes to human health [1]. Diverse tomato germplasm accessions carrying abundant genetic variations are widely cultivated all over the world. Unraveling the origin and functionality of genetic variants is central to exploring complex agronomic traits in tomato, as these natural alleles can be used to guide tomato breeding efforts [2]. Thus far, numerous tomato germplasm resources have been collected and analyzed [2, 3]. The use of genomic resources, ranging from linear to pangenomes [3–8], coupled with multidimensional datasets [9, 10] has revealed similarities and differences within species and provided candidate natural alleles for modeling the genetic architectures of agronomic traits. However, comprehensively capturing the causal variations that underlie the trait heritability is a challenging endeavor, as most agronomic traits targeted for tomato improvement are controlled by multiple loci [3]. Therefore, an extensive, precise catalog of variations from multiple dimensions (e.g. genomics, variomics, and metabolomics) is required to discover highly reliable alleles for tomato breeding.

Germplasm resources are essential for identifying genes associated with agronomic traits. In tomato, several genes influencing fruit weight (FW), quality, and disease resistance have been characterized in natural populations using multi-omics technology [11, 12]. Nonetheless, the simplicity of the linear reference genome and the limitations of short-read sequences hinder the identification of causal genetic variants. Therefore, the genetic relationships between complex traits and causal variants remain elusive. Recently, graph-based pan-genomics approaches have enabled more thorough and accurate detection of structural variants (SVs) and have greatly facilitated the identification of natural alleles [3]. However, focusing solely on SVs may limit our ability to capture the trait heritability, as single nucleotide polymorphisms (SNPs) and insertions and deletions (INDELs) may be equally important in revealing the genetic mechanisms underlying agronomic traits [13]. Accurately integrating all genetic variants into a single genome graph is highly challenging due to limitations in computational resources and power [14]. To overcome these bottlenecks, it is essential to construct a genetic population that captures the full spectrum of genetic variation and to analyze the interactions and regulatory mechanisms of these variants using a graph-based pangenome.

Here, we developed a graph-based pangenome that encompasses the complete variation profile (SNPs, INDELs, and SVs) from the wild and cultivated tomato parents and their F_7_ recombinant inbred line (RIL) population. Based on this integrated framework, genomic, metabolomic, and functional analyses delineated the core genetic architecture underlying multiple agronomic traits in tomato (Fig. S1).

## Results

### Establishment of recombinant inbred lines

**Figure 1:**
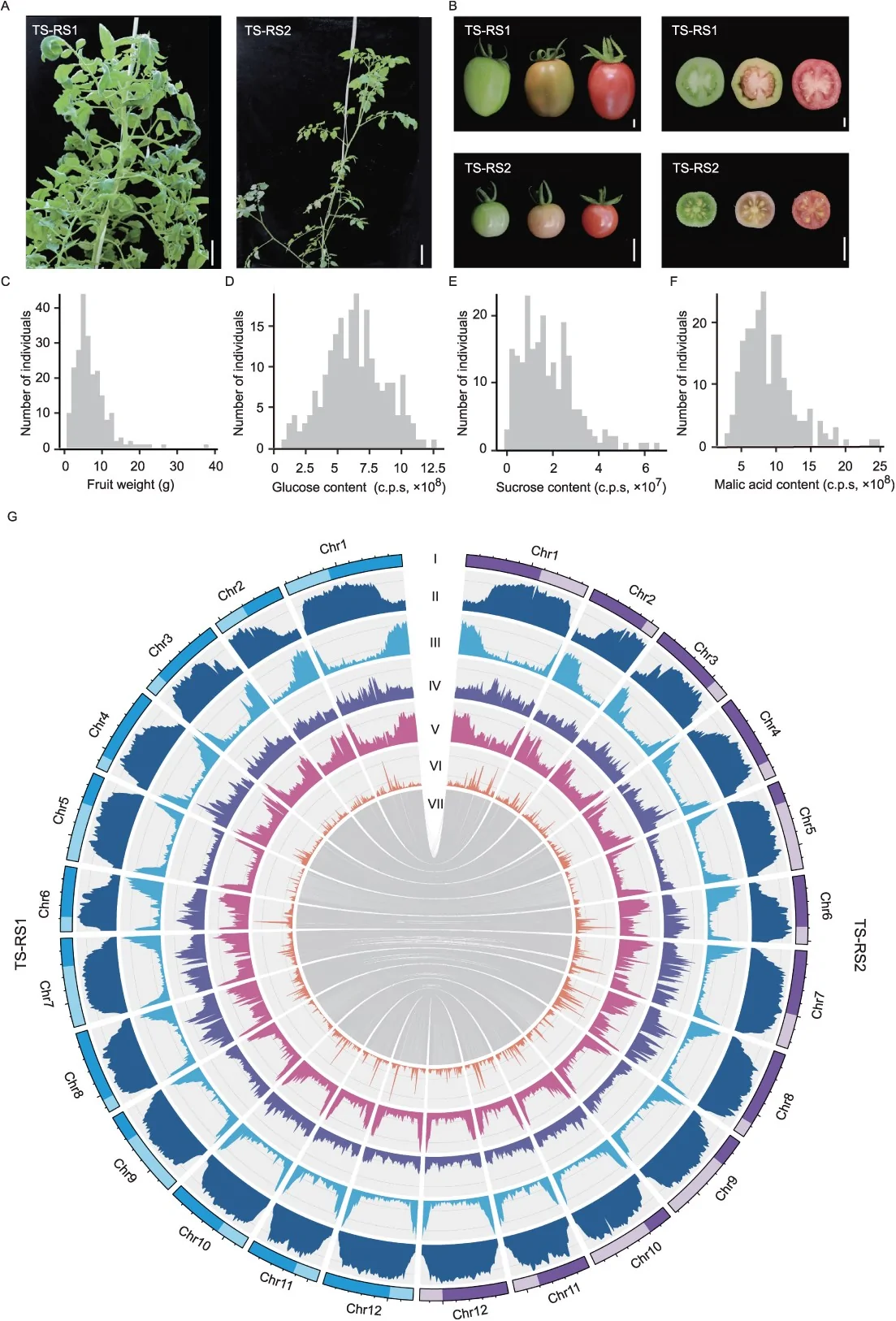
Morphology and genomic features of *S. lycopersicum* (TS-RS1) and *S. pimpinellifolium* (TS-RS2). (A and B) Whole plants (scale bars: 5 cm) (A) and fruits (scale bars: 1 cm) (B) of TS-RS1 and TS-RS2. (C–F) Distribution of FW (C), glucose (D), sucrose (E), and malic acid (F) content in the F_7_ RIL population. c.p.s., counts per second. (G) Ideogram of the chromosomes (i), Repeat content (ii), Gene density (iii), SNPs density (iv), INDELs density (v), SVs density (vi), and collinear blocks (vii) of TS-RS1 and TS-RS2.

To precisely map the genetic loci controlling FW, vegetative form, trichome, and quality-related metabolism, it is necessary to select parental lines that exhibit significant phenotypic divergence in these traits and to construct an RIL population from them. We examined various phenotypic traits in 227 tomato accessions comprising two phylogenetic groups [*Solanum pimpinellifolium* (PIM) and *S. lycopersicum* (BIG)] (Fig. S2). Aside from the differences arising from the distinct genetic backgrounds [2], two key agronomic traits, FW and soluble solid content (SSC), differed significantly between the PIM and BIG accessions. Specifically, six PIM accessions exhibited small fruits (FW ≤ 4 g) and high SSC (≥6.61%), while 69 BIG accessions showed large fruits (FW ≥ 32 g) and low SSC (≤4.05%) (Table S1). Among these accessions, we discovered the red large-fruited BIG accession TS-RS1 with the determinate growth and trichomes, which has been widely used in tomato disease resistance studies [15], and the wild PIM species TS-RS2 that has desirable aromas and rich fruit flavor, along with the indeterminate growth and the lowest genomic heterozygosity (Fig. 1A and B and Table S1). Accordingly, we constructed a RIL population derived from these two accessions.

We crossed the TS-RS1 and TS-RS2 to generate an F_7_ RIL population consisting of 230 individuals. The RIL population exhibited typical phenotypes with genetic segregation, including FW (0.73–32.00 g), glucose (9.71 × 10^7^–1.27 × 10^9^ c.p.s.), sucrose (6.11 × 10^5^–6.59 × 10^7^ c.p.s.), and malic acid (2.78 × 10^8^–2.44 × 10^9^ c.p.s.) contents (Fig. 1C–F). These data indicated that abundant genetic variation exists in this RIL population, enabling the dissection of genetic mechanisms underlying agronomic traits. Importantly, both parental lines possessed high-quality genome assemblies with low heterozygosity rates (<0.03%). Therefore, we selected the biparental accessions and their segregating population to construct a graph-based pangenome to reveal the genetic mechanism of agronomic traits of tomato fruits.

### Construction of reference-level genomes of the biparental accessions

To obtain a comprehensive catalog of genetic variants in the RIL population, we sequenced the genome of two parental accessions (TS-RS1 and TS-RS2) and generated 121.6 and 76.4 Gb (151.0 and 91.5×) of PacBio long reads, 191.9 and 207.3 Gb (127.0 and 112.6×) of BioNano optical genome mapping data, 28.1 and 30.0 Gb (35.7 and 36.7×) of Hi-C data, and 51.3 and 34.4 Gb (64.0 and 41.7×) of Illumina short reads, respectively (Table S2). Two genomes were assembled *de novo*, yielding total genome sizes of 805.54 and 835.42 Mb comprising 12 pseudo-chromosomes, with contig N50 size of 36.34 and 19.83 Mb (Table 1). We then sequenced the mRNA from five representative tissues of both accessions and annotated 34 544 and 33 379 protein-coding genes across the TS-RS1 and TS-RS2 genomes, of which 97.00% and 98.58% were anchored to 12 chromosomes, respectively (Fig. 1G and Table 1). In addition, approximately 553.88 and 578.45 Mb (68.76% and 69.24%) of repetitive sequences were identified for TS-RS1 and TS-RS2. Long terminal repeats (LTRs) were the major repetitive sequences, covering 51.47% (414.63 Mb) and 50.74% (423.89 Mb) of the TS-RS1 and TS-RS2 genomes, respectively. Among these, the *Gypsy*- and *Copia*-types were the most abundant. They accounted for 43.35% (349.20 Mb) and 7.16% (57.70 Mb) of the TS-RS1 genome, in contrast to 42.82% (357.69 Mb) and 6.90% (57.66 Mb) of the TS-RS2 genome (Table S3). Moreover, the Benchmarking Universal Single-Copy Orthologue (BUSCO) scores were ~97% for both genomes, and the LTR Assembly Index values were approximately 14 (Table S4), demonstrating the high completeness and coverage of both genome assemblies.

**Table 1:** Global characteristics of the TS-RS1 and TS-RS2 genomes.

**Statistics**	**TS-RS1**	**TS-RS2**
Assembly size (Mb)	805.54	835.42
Number of contigs	711	722
Mean contig (bp)	1 130 089	1 149 339
Contig N50 (bp)	36 337 478	19 826 020
Contig N90 (bp)	25 516 074	16 423 166
Largest contig (bp)	47 148 369	31 452 562
Number of genes	34 544	33 379
Mean gene length (bp)	3327.92	3424.08
Number of CDSs	156 145	153 475
Mean CDS length (bp)	233.07	231.39
Number of exons	156 145	153 475
Mean exon length (bp)	233.07	231.39
Repetitive sequences (Mb)	553.88	578.45
Repetitive percentage (%)	68.76%	69.24%

**Figure 2:**
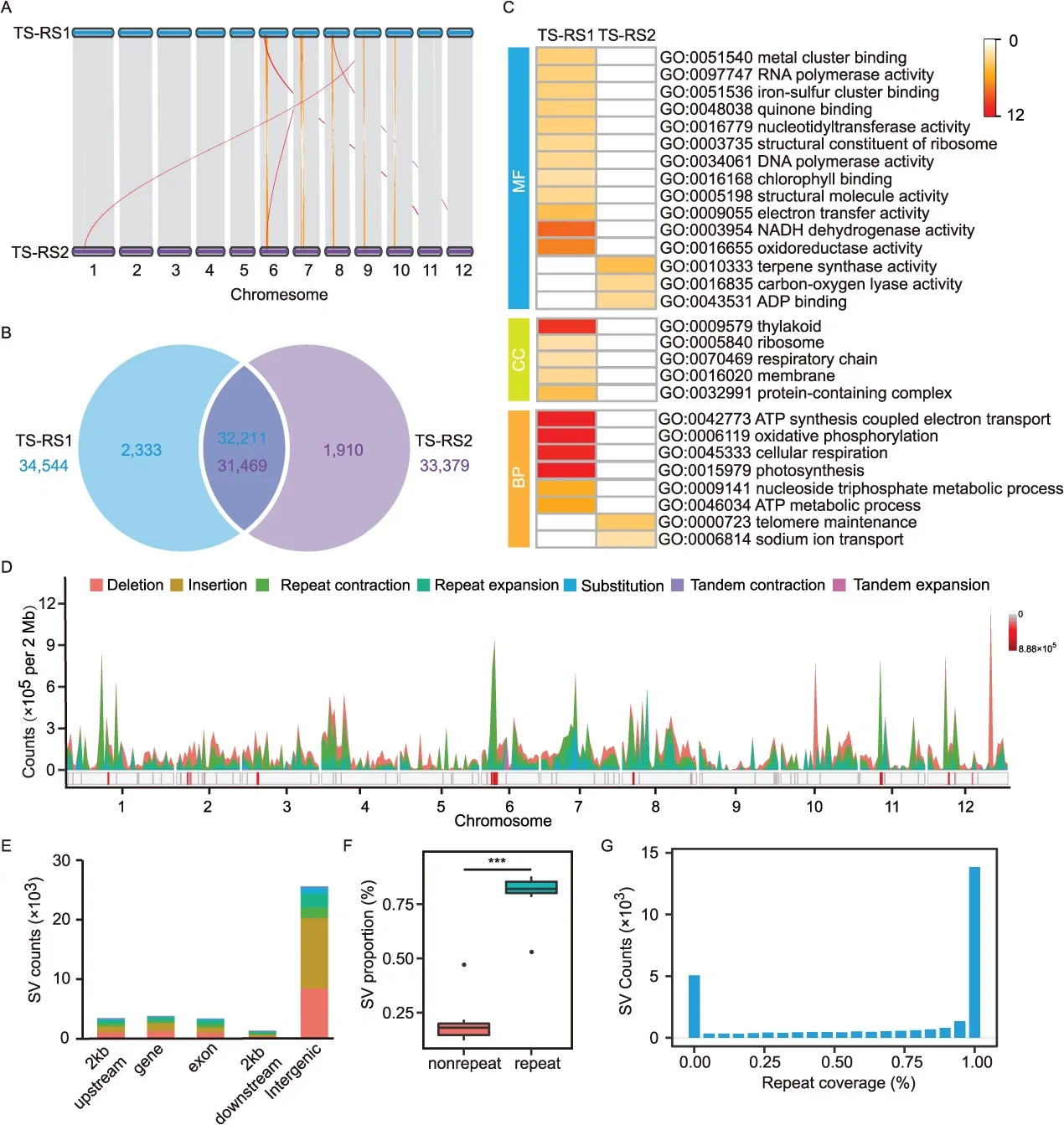
Genomic comparison of *S. lycopersicum* (TS-RS1) and *S. pimpinellifolium* (TS-RS2). (A) Genome collinearity features between TS-RS1 and TS-RS2. (B) Venn diagrams of the number of collinearity and noncollinearity genes. (C) GO enrichment analysis of TS-RS1 and TS-RS2 genomes specific genes. (D) The number of uniquely located SVs at different regions in the TS-RS1 genome, counted in 2-Mb windows. The color bar represents the centromere density, defined as the total length of centromeric sequences (bp) per 1 Mb sliding window. (E) The number of SVs overlapping different genomic features. (F) Structural variation density in repeat/non-repeat genome regions. Statistical significance was determined by a two-sided Student’s *t*-tests (^***^P < 0.001). (**G**) Structural variation number plotted against repetitive DNA coverage.

We compared the genomic features of the wild species TS-RS2 with those of the cultivated tomato TS-RS1 and found that 28 151 (84.34% of *TS-RS1* genes) and 28 107 (81.37% of *TS-RS2* genes) were collinear between two genomes (Fig. 2A). Moreover, we identified 2333 and 1910 genes that were specific to TS-RS1 and TS-RS2, respectively, possibly owing to the high sequence divergence that arose during tomato domestication (Fig. 2B). Gene Ontology (GO) analysis of specific genes showed significant enrichment (*P* < 0.01) for terms including ‘NADH dehydrogenase activity’, ‘oxidoreductase activity,’ and ‘terpene synthase activity’ (Fig. 2C). Furthermore, we observed 97.01% synteny between the TS-RS1 and TS-RS2 genomes (Fig. 1G), and cataloged a total of 5 263 881 SNPs, 734 929 INDELs, and 28 333 SVs (Table S5). Within the syntenic genomic regions, 3925 and 3846 genes were affected by 3753 and 3635 SVs in the TS-RS1 and TS-RS2 genomes, respectively (Table S6). Of these genes, only 11.72%–12.52% underwent human selection [2], including 1207 domesticated genes and 872 improved genes. We found SVs were distributed throughout the genome, with a notable enrichment in the centromeric regions (Fig. 2D), indicating the presence of SV hotspots is located near these regions. These SVs are predominantly located within intergenic regions, and when they occur within genes, they most frequently affect the coding regions (Fig. 2E). We also observed a much higher proportion of SVs (79.10%) in repetitive regions (Fig. 2F and G). The predominant repeats harboring SVs were *Gypsy*-type LTRs, accounting for 7.73% of the TS-RS1 genome. We additionally identified 3476 and 2445 INDELs that caused frameshift mutations and other potentially deleterious protein-coding variants, affecting 2162 and 1534 genes in the TS-RS1 and TS-RS2 genomes, respectively (Table S6). Our data provide a comprehensive variant set for constructing the graph-based pangenome, offering a rich source of natural alleles for additional functional genomics studies and breeding opportunities in tomato.

### Construction of the graph-based pangenome

**Figure 3:**
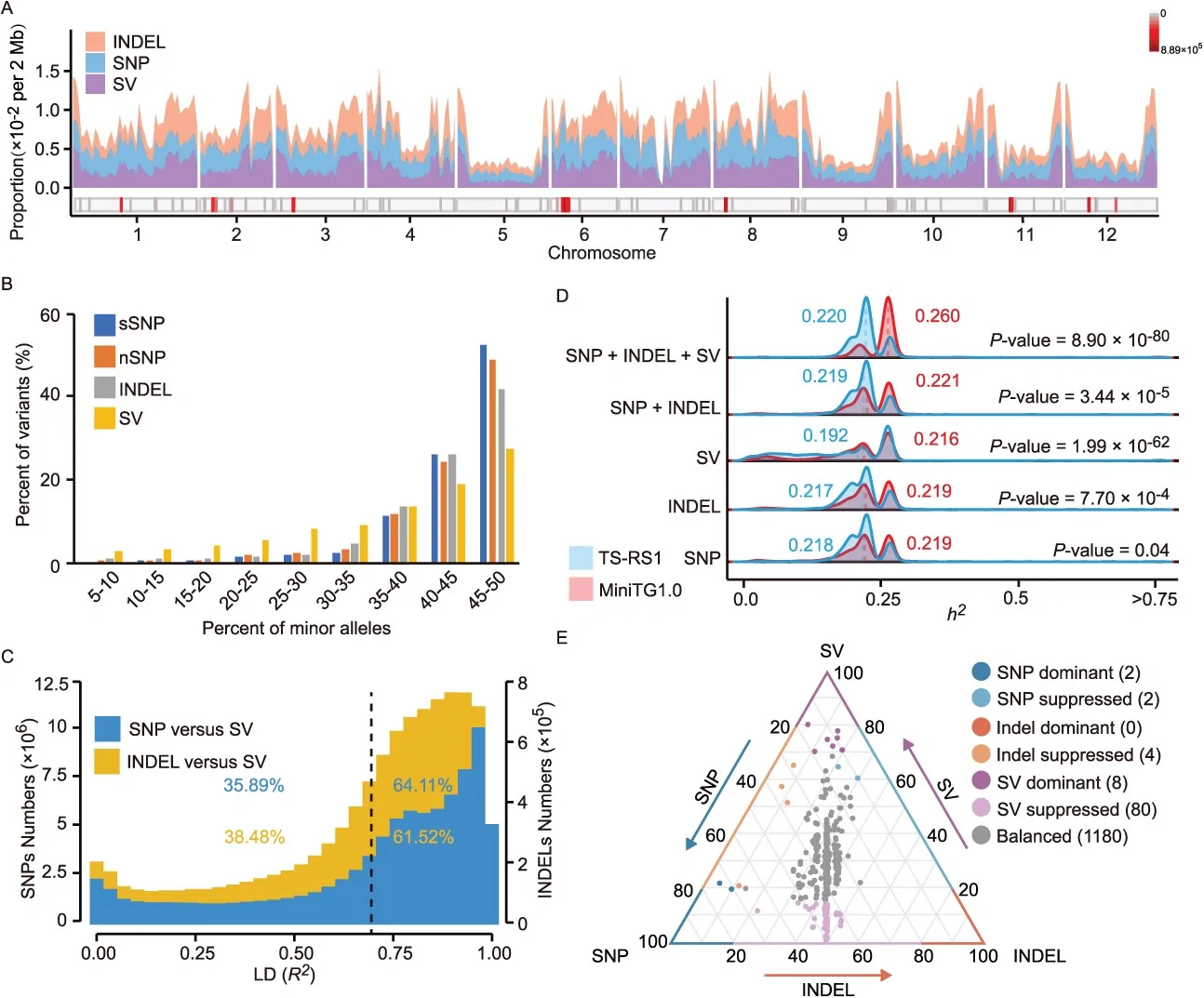
Genetic variations in the MiniTG1.0 of the RIL population. (A) Graph-based pangenome identification of the proportion of variations located in different regions of a 2-Mb window of the TS-RS1 genome. The color bar represents the centromere density, defined as the total length of centromeric sequences (bp) per 1 Mb sliding window. (B) MAF for synonymous (sSNP), nonsynonymous (nSNP), INDEL, and SV. (C) Distribution of LD (*R*^2^) between SVs and SNPs or INDELs within 50 kb of SVs. The dashed line indicates *R*^2^ = 0.70. (D) Comparison of heritability (*h*^2^) estimated using different combinations of genetic variants in the MiniTG1.0 and the TS-RS1 linear genome. (E) Proportion of the heritability of SNPs, INDELs, and SVs contributing to traits.

Using the TS-RS1 genome as the reference, we constructed a nearly comprehensive graph-based pangenome, including all SNPs, INDELs, and SVs identified from both parents. The resulting graph-based pangenome (MiniTG1.0) spans 904.65 Mb and contains 36 747 protein-coding genes, of which 2203 were detected within the 113.20 Mb of sequence derived from the TS-RS2 genome. To ensure sufficient power for the genetic analysis of the entire population, we resequenced a total of 230 RILs, generating ~1.70 Tb of sequence data. We identified 5 761 554 variants in the RIL population (Fig. 3A). Comparison of the variant composition between the parental lines and the RILs revealed that 99.65% of the variants identified in the RIL population were inherited from the parents (Fig. S3A). Among these, 9377 variants were newly identified in the RIL population, of which 3913 were SVs. The mean minor allele frequency (MAF) of these SVs was 0.23, significantly lower than that of the overall SV set (Fig. S3B). Three randomly selected structural variations were experimentally validated in the RIL population (Fig. S3C). We also identified 5 569 512 variants in the linear TS-RS1 genome. To construct a high-density tomato genetic map, we employed a Hidden Markov Model to infer all recombination events in the RIL population. We identified 10 682 recombinant fragments, which were used as bin markers, spanning a total length of 790.61 Mb and covering 99.89% of the TS-RS1 genome (Fig. S4A). Collinearity analysis revealed that the order of most markers on each linkage group was consistent with their genomic positions (Fig. S4B). These results demonstrate that the graph-based pangenome (MiniTG1.0) captures a comprehensive landscape of sequence variations segregating within the RIL population.

Next, we investigated the allele frequency distributions to evaluate the potential impact of these variants. For SNPs, INDELs, and SVs, the MAF was largely centered near 50% (Fig. 3B). Here, we found that 35.89% and 38.48% of SVs were in low linkage disequilibrium (LD) with adjacent SNPs and INDELs (50 kb; *R*^2^ < 0.7) (Fig. 3C). The heritability of agronomic traits explained by genetic variants is crucial for gene pyramiding in crop breeding. Based on the resulting genotypes, we estimated the power of the MiniTG1.0 in capturing the missing heritability of 1276 characteristic phenotypes, comprising 18 morphological traits and 1258 metabolic traits. SNP and INDEL heritability were significantly greater in the MiniTG1.0 compared to the linear TS-RS1 genome (*P* < 0.05; Fig. 3D). Intriguingly, higher SV heritability was detected in the MiniTG1.0 (0.216) than in the linear TS-RS1 genome (0.192; *P* = 1.99 × 10^−62^). Furthermore, joint analysis of all variants showed that the average heritability in the MiniTG1.0 was 0.260, which is 18.18% higher than that of the linear TS-RS1 genome (*P* = 8.90 × 10^−80^) and represents a 1.19-fold increase over the heritability obtained from each individual variant callset (Fig. 3D). In addition, the share of heritability for 1180 phenotypes was balanced among SNPs, INDELs, and SVs (1180 of 1276; 92.25%) (Fig. 3E). These findings suggest that we should identify abundant genetic variants and more accurately determine the heritability of agronomic traits in used MiniTG1.0.

### Identification of the loci related to trichome density, growth habit, and fruit weight

To decipher the genetic basis underlying key agronomic traits in tomato, we performed genome-wide association studies (GWAS) for multiple traits, including trichome density, growth habit, and FW using the MiniTG1.0. For the trichome (*h*^2^ = 0.76), we identified a C2H2 zinc finger protein (encoded by the *H* gene) within the bin9678 on chromosome 10. Previous studies have reported that silencing this gene leads to trichome deficiency [16]. Notably, we further detected a 4333-bp SV (SV*_H_*) that likely affects the presence or absence of the *H* gene (*TLT01_Solyc10G019190*) (Fig. S5B). However, when the linear genome was used, the significant SNP signal (SNP*_H_*, TS-RS1:Chr10:61553231) was mapped to bin9676, approximately 67.60 kb downstream of the *H* gene, within a genomic interval spanning nine genes (Fig. S5A and C). In the GWAS for vegetative form, we observed a discrepancy in results consistent with the trichome traits. The lead SNP identified using the linear reference genome and that mapped with the MiniTG1.0 resided in distinct bin markers (Fig. S5D and E). Notably, the MiniTG1.0 precisely localized the major signal to bin5518 on chromosome 6, which colocalizes with the genomic interval of *SELF-PRUNING* (*SP*) [17], a key gene known to control tomato vegetative form (*h*^2^ = 0.87) (Fig. S5F). These results suggest that the MiniTG1.0 exhibits higher accuracy than the linear genome in identifying the candidate genes associated with agronomic traits.

**Figure 4:**
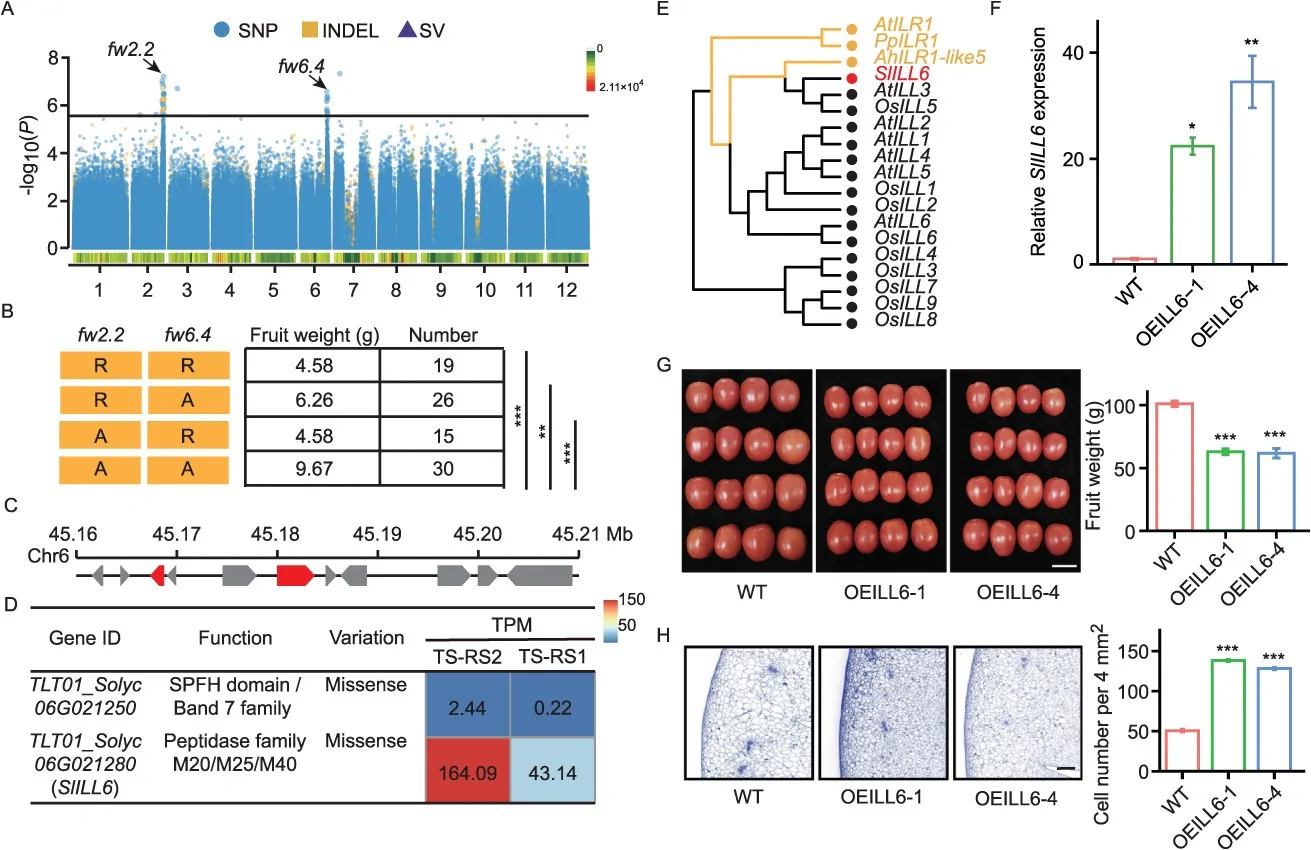
The *fw2.2* and *fw6.4* locus modulates tomato FW. (A) Association studies of FW using the GWAS. (B) Allele distribution in varieties containing R or A allele combinations associated with FW. R is the wild TS-RS2 allele and A is the cultivated TS-RS1 allele. (C) The distribution of genes in the *fw6.4* locus. (D) Expression and variation of two candidate genes in the fruit of different genotypes. NA, no annotated information. (E) Phylogenetic tree of *SlILL6* and its homologs in rice (Os), Arabidopsis (At), peach (Pp), peanut (Ah), and tomato (Sl). (F) The relative expression levels of *SlILL6* in the overexpression lines. (G) Fruit morphologies and weight of WT and two overexpression lines. Data are presented as mean ± SEM (*n* = 16). Scale bar: 5 cm. (H) Sections of fruit tissues in wild and overexpression lines. Data are presented as mean ± SEM (*n* = 3). Scale bar: 2 mm. Statistical significance was determined by two-sided Student’s *t*-tests (^**^P < 0.01; ^***^P < 0.001; n.s., not significant).

We sought to identify the genes associated with FW (*h*^2^ = 0.82) and identified two significant signals using MiniTG1.0. The known *fw2.2* on chromosome 2 (TS-RS1:Chr2:54238042, *P* = 6.53 × 10^−8^) and a novel locus on chromosome 6 (TS-RS1:Chr6: 45161479, *P* = 2.80 × 10^−7^), which we designated *fw6.4* (TS-RS1:Chr6:45157964-45217 136) (Fig. 4A). Haplotype analysis revealed that the RR genotype at these two FW loci is associated with smaller fruit size (Fig. 4A), suggesting they may interact synergistically or additively to influence the trait. Further analysis of *fw6.4* indicated that the genomic region under the association signal contains 11 protein-coding genes, two of which exhibited lower expression levels in cultivated tomato TS-RS1 (Fig. 4C and D). *IAA-LEU-RESISTANT1 (ILR1)-LIKE 6* (*SlILL6*, *TLT01_Solyc06G021280*), encoding an indole-3-acetic acid-amino acid hydrolase, resides 57.74 kb downstream of the strongest association signal. The orthologs of this gene include *AtILR1*, *AhILR1-LIKE5*, and *PpILR1*, which respectively regulate *Arabidopsis* (*Arabidopsis thaliana*) seedling development [18], peanut (*Arachis hypogaea*) seed size [19], and peach (*Prunus persica*) fruit ripening [20] (Fig. 4E). Moreover, *SlILL6* had a higher expression level in small-fruited individuals than in the big-fruited individuals during fruit ripening (Fig. S6). To determine whether *SlILL6* is the causative gene governing FW, we evaluated two *SlILL6-*overexpressing lines during fruit ripening (Fig. 4F) and observed lower FW in the overexpression lines (62.96 ± 2.31 g in OEILL6-1 and 61.82 ± 3.75 in OEILL6-4) than in the wild-type line (WT) (101.12 ± 2.33 g) (Fig. 4G). In addition, the fruit of two overexpressing lines exhibited significant differences in the transverse and longitudinal dimensions compared to the WT line (Fig. S7). Microscopic observation showed that the thin-walled cortical cells in *SlILL6*-overexpressing fruit (133.33 ± 2.39) were significantly smaller than those in WT (50.67 ± 1.67) (Fig. 4H). Collectively, these findings demonstrate that *SlILL6* negatively regulates FW by affecting cell size in tomato fruit, highlighting this gene as a potential target for manipulating fruit size in breeding programs.

### The metabolic atlas of tomato fruit ripening

**Figure 5:**
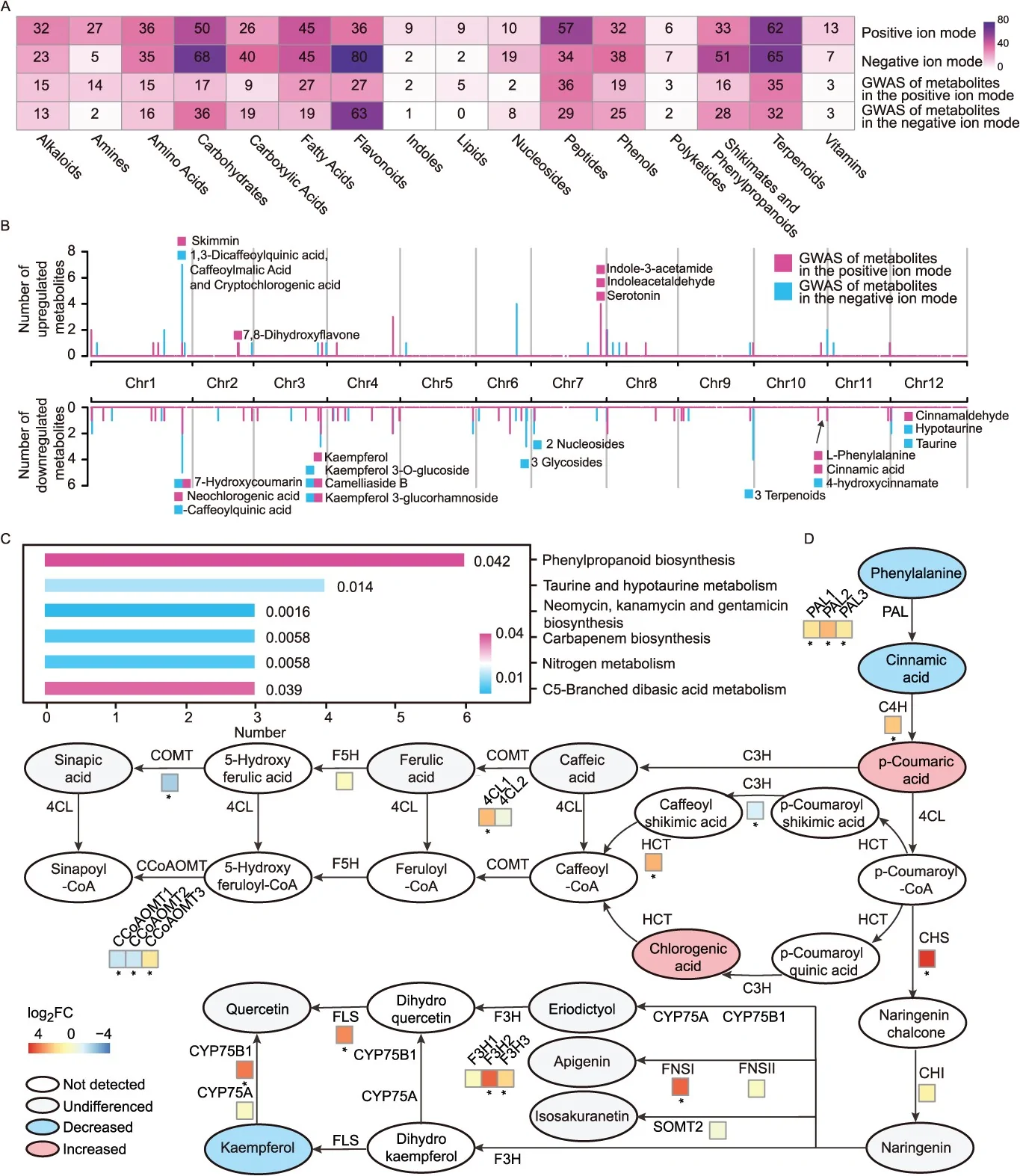
Mapping of metabolic QTLs across the RIL population using GWAS. (A) The number of metabolites in 16 metabolic groups, together with those for which significant association signals were detected. (B) The number of differentially accumulated metabolites per bin marker. (C) KEGG enrichment analysis of differentially accumulated metabolites. (D) The phenylpropanoid biosynthesis. Heatmap showing the expression levels of each gene between TS-RS1 and TS-RS2 (TPM values). Asterisks (*) indicate DEGs.

The composition and abundance of metabolites collectively shape the flavor profile, nutritional quality, and postharvest disease resistance of tomato fruits. To pinpoint the metabolic changes that occur during fruit ripening, we analyzed the metabolome of fruits harvested at the turning stage. We selected 230 individuals and quantified fruit metabolites using a U3000 Ultra-High-Performance Liquid Chromatography (UHPLC) system coupled with a Q-Exactive Orbitrap mass spectrometer. Under both positive and negative ion modes, a total of 1258 metabolites were detected and annotated, which were then categorized into 16 groups based on their structure and associated pathways (Fig. 5 and Table S7). Heritability (*h*^2^) estimates revealed that 94.20% (1185) of these metabolites exhibited values exceeding 0.2. Additionally, 98.01% (1233) of the metabolites exhibited a coefficient of variation (CV) greater than 0.2 (Fig. S8). To identify key genes and loci regulating fruit metabolic traits, we performed a metabolome-based GWAS (mGWAS) on the 230 individuals in the RIL population. A total of 49 907 significant association signals for 651 metabolites were identified, covering 535 bin markers on 12 chromosomes using the Mixed Linear Model (MLM) [21] (Fig. 5A). Among these association signals, 128 metabolites showed differential accumulation in TS-RS1, with 78 upregulated and 50 downregulated, potentially reflecting selection on fruit mass during domestication and improvement (Fig. 5B). Notably, metabolites sharing structural similarities or biosynthetic pathways were frequently co-localized within the same genomic regions of the MiniTG1.0, such as six caffeoyl analogs (TS-RS1:Chr1:82216475-82377690), three glycosides (TS-RS1:Chr6:45305578-45531454), and four auxins (TS-RS1:Chr7:62919836-63172842).

Based on the Kyoto Encyclopedia of Genes and Genomes analysis, the differentially accumulated metabolites are significantly enriched in phenylpropanoid biosynthesis, nitrogen metabolism, ubiquinone and other terpenoid-quinone biosynthesis, and lysine and histidine metabolism (Fig. 5C), which may be associated with fruit quality and stress tolerance [9]. Additionally, five differentially accumulated metabolites are associated with phenylpropanoid biosynthesis, namely, phenylalanine, cinnamic acid, *p*-coumaric acid, kaempferol, and chlorogenic acid. We compared the expression patterns of genes related to the biosynthesis of these metabolites in the parental lines. From this comparison, we detected 16 differentially expressed genes (DEGs), including *PAL*, *C4H*, *4CL*, *CHS*, *C3H*, *HCT*, *COMT*, *CCoAOMT*, *FNSI*, *F3H*, *FLS*, and *F3′H* (*CYP75B1*) (Fig. 5D). Significant correlations were observed among the contents of phenylalanine (*h*^2^ = 0.73, CV = 0.46), cinnamic acid (*h*^2^ = 0.54, CV = 0.47), and *p*-coumaric acid (*h*^2^ = 0.22, CV = 1.19) in the RIL population (Fig. S9A). mGWAS of these metabolites co-localized their significant association signals to bin9828 at the end of chromosome 10, a genomic interval containing the phenylalanine ammonia-lyase (*PAL*) gene (Fig. S9B). Further analysis of allelic variation in *PAL* revealed significant differences in metabolite content among individuals with different genotypes (Fig. S9C–E). Moreover, kaempferol and its three glycosylated derivatives (*h*^2^ = 0.20–0.77, CV = 0.81–1.06) showed a highly positive correlation in their accumulation patterns (Fig. S10A). Their mGWAS signals were co-enriched within the same bin marker interval on chromosome 3, which harbors an annotated flavonoid-3′-hydroxylase (*F3′H*) gene (Fig. S10B). Genotyping of *F3′H* revealed that accessions carrying different allelic variants exhibited significant differences in the content of these compounds (Fig. S10C–F). Many genetic loci controlling phenylpropanoid metabolism in ripening tomato fruits were identified from MiniTG1.0, which will facilitate the improvement of tomato fruit quality.

### A GDSL lipase-like caffeoyltransferase governs chlorogenic acid-mediated disease resistance

**Figure 6:**
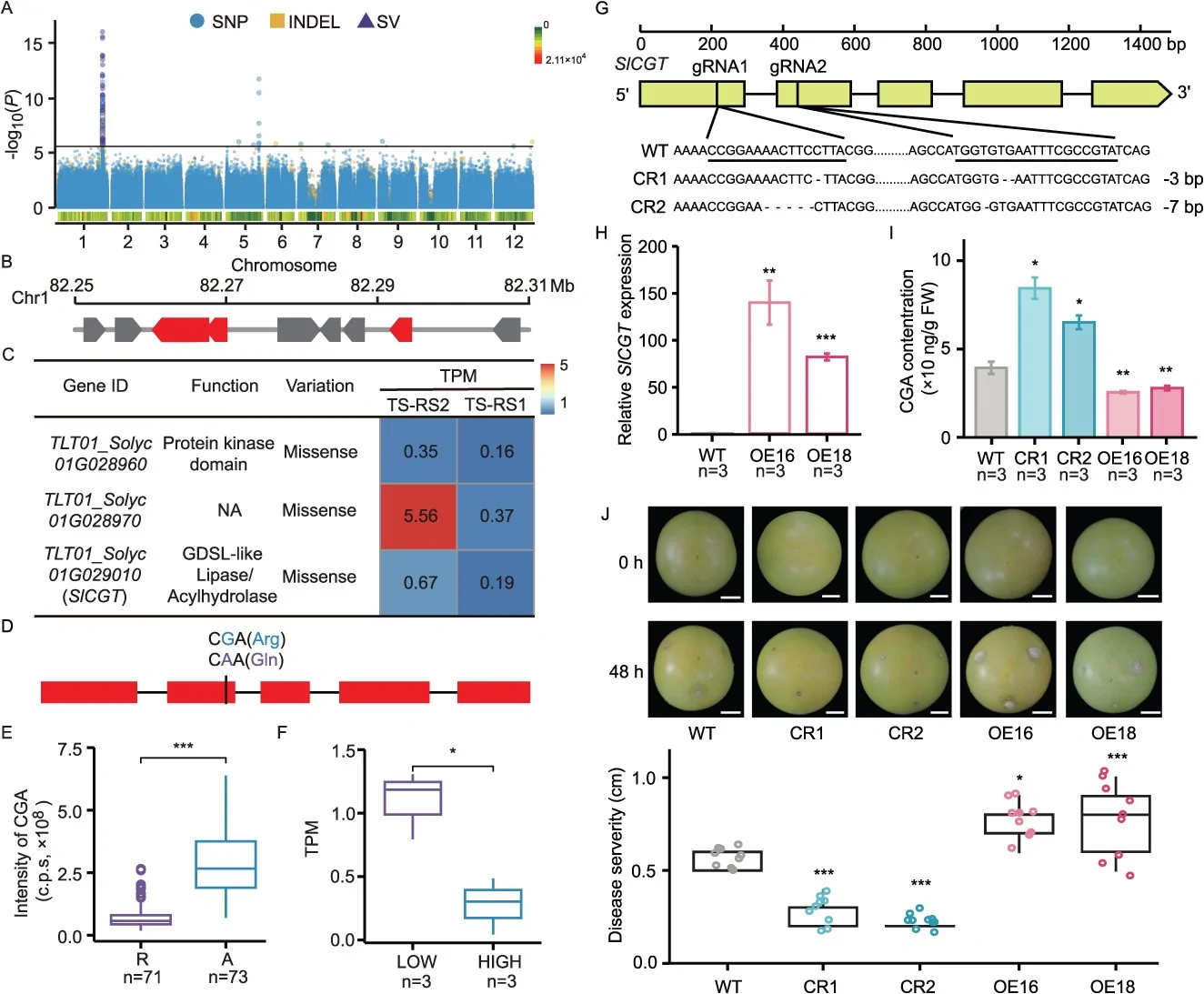
Characterization of the GDSL lipase regulating chlorogenic acid content. (A) Manhattan plots of GWAS for CQA. (B) The distribution of genes in the signal. (C) Expression and variation of three candidate genes in the fruits of different genotypes. NA, no annotated information. (D) Gene structure of *SlCGT*. (E) Allele distribution in varieties containing R or A allele combinations associated with CQA. R is the wild TS-RS2 allele and A is the cultivated TS-RS1 allele. (F) *SlCGT* expression in fruits of RIL accessions with high and low chlorogenic acid content (*n* = 3). (G) Genomic sequence showing the gRNA target site and the resulting CRISPR/Cas9-induced mutations in *SlCGT* in the knockout lines (CR1 and CR2). (H) The relative expression levels of *SlCGT* in the overexpression lines (*n* = 3). (I) Comparison of the CQA content between the WT and the *SlCGT* transgenic lines (data are presented as mean ± SEM, *n* = 3). (J) Lesion diameter in turning-stage fruits of the *SlCGT* transgenic lines compared to WT 48 h after *B. cinerea*-inoculation (*n* = 9). The correlation calculation uses the spearman correlation coefficient, and statistical significance was determined by two-sided Student’s *t*-tests (^*^P < 0.05; ^**^P < 0.01; ^***^P < 0.001).

Phenylpropanoids are the core metabolites influencing fruit quality and play essential roles in plant disease resistance [22]. Chlorogenic acid (CQA), an important phenylpropanoid, exhibits strong antioxidant activity in plants [23]. We performed GWAS for CQA (*h*^2^ = 0.75, CV = 0.80) and identified one significantly association signal (TS-RS1:Chr1:82321256; *P* = 1.11 × 10^−16^) at the distal end of chromosome 1 (Fig. 6A). We narrowed the interval to a ~72.65-kb region (bin1358) containing nine putative protein-coding genes (Fig. 6B). Based on transcriptomic analysis, three differentially expressed genes were pinpointed as candidates (Fig. 6C). Causal variations analysis highlighted one domesticated gene, GDSL lipase-like caffeoyltransferase (*SlCGT*, *TLT01_Solyc01G029010*) as the key candidate gene for chlorogenic acid biosynthesis. A nonsynonymous mutation in *SlCGT* is significantly associated with elevated metabolite levels in cultivated varieties (Fig. 6D and E). Furthermore, *SlCGT* expression differed significantly between RILs with high *versus* low CQA content (Fig. 6F). These findings indicate a potential role for *SlCGT* in negatively regulating chlorogenic acid accumulation.

To determine the function of *SlCGT*, we generated knockout lines by clustered regularly interspaced short palindromic repeats-associated nuclease 9 (CRISPR-Cas9)-based genome editing and overexpression lines in the cultivated tomato cv. Ailsa Craig (Fig. 6G and H). Compared to WT, the total CQA content was significantly lower in the overexpression lines and higher in the knockout lines (Fig. 6I). However, these alterations did not negatively impact fruit SSC (Fig. S11). As previously reported, chlorogenic acids play important roles in plant disease resistance [24]. CQA exerted direct antifungal activity against *Botrytis cinerea in vitro* (Fig. S12A). This protective effect was confirmed *in vivo*, where applying 200 μg/ml CQA to tomato fruits effectively controlled gray mold (Fig. S12B). To directly test whether *SlCGT* expression responds to fungal infection, we inoculated fruits at the turning stage from knockout and overexpression lines with *B. cinerea*. As expected, lesion diameters on the overexpression fruits were significantly greater compared to the WT, whereas lesion diameters were smaller in the knockout lines (Fig. 6J). Our findings suggest that *SlCGT* negatively regulates chlorogenic acid-mediated disease resistance, offering insights for harnessing this pathway to bolster plant defense and improve fruit quality.

## Discussion

Comparative genomics analysis can be used to explore evolutionary events, identify orthologous sequences, uncover genes related to agronomic traits, and detect large differential segments among genomes. State-of-the-art graph pangenomes open new avenues for incorporating more SVs from the natural populations into cultivated species [3, 13, 25–29]. In tomato, the graph pangenome captured the missing heritability of 20 323 molecular traits, including the expression of 19 353 genes and 970 metabolite traits [3]. The super-pangenomes of nine wild and two cultivated species revealed the evolutionary history of tomato and enabled the discovery of wild alleles through the detection of SVs [5]. However, despite these advances, current graph-based pangenome frameworks often prioritize SVs and overlook the integration of smaller genetic variants such as SNPs and INDELs [3, 13, 26]. This limitation restricts the comprehensive detection of trait-associated variations and undermines the power of pangenomes for precise gene mapping and breeding applications. In this study, we developed a graph-based pangenome in tomato (MiniTG1.0) to facilitate improved gene mapping and polymerization breeding. By constructing MiniTG1.0, a genome that integrates all variants from both parents, we were able to perform a comprehensive analysis of genetic variation and explore the mechanisms regulating these traits. Our graph-based pangenome integrated the full repertoire of genetic variants (SNPs, INDELs, and SVs) within a streamlined topology, enabling high-efficiency and high-precision population-genetic studies. As expected, the vast majority (99.65%) of variants identified in the RIL population were inherited from the two parental lines, confirming the genetic integrity of our population. However, we also detected a small number of variants present in the RIL population but absent in the parental lines, of which 41.73% were SVs. These newly identified SVs exhibited significantly lower minor allele frequencies, and their authenticity was partially validated through resequencing data. This observation suggests that *de novo* mutations may have occurred during the selfing process of the RIL population [30].

Another strong advantage of the graph-based pangenome approach is our use of the RIL population derived from a cross between the cultivated accession *S. lycopersicum* (TS-RS1) and the wild species *S. pimpinellifolium* (TS-RS2). The parents exhibit obvious phenotypic differences and are considered potential germplasms for human selection. The high frequency of recombinant events observed in this RIL population enabled the construction of a high-resolution variation map within MiniTG1.0, significantly enhancing its power for precise gene-trait association studies [31, 32]. Here, we conducted a comprehensive study of sequence variations in MiniTG1.0 and metabolic alterations detected by mGWAS. We successfully identified genes related to FW and metabolites using the multi-omics data from the RIL population. Our findings emphasize that, despite advances in pangenome resources, the strategic use of purposefully designed populations remains critical for functional genomic studies in tomato.

Fruit quality is typically targeted by human selection in crops such as apple (*Malus domestica*), watermelon (*Citrullus lanatus*), and loquat (*Eriobotrya japonica*) [33–35]. In tomato, several FW-associated genes, such as *fw2.2*, *fw2.3*, and *fw11.3*, have been identified as targets of human selection [2]. Nevertheless, a considerable number of genetic loci governing FW remain uncharacterized. In this study, genome-wide association analysis (GWAS) integrated with genetic and molecular evidence successfully identified two loci significantly associated with FW: the known major-effect gene *fw2.2* [36] and a novel locus, *fw6.4*. Within the *fw6.4* locus, *SlILL6* likely acts as a key gene that negatively regulates FW by influencing fruit cell expansion. While research in *Arabidopsis* links the *ILR* gene family to the regulation of auxin and other metabolites [37–39], the mechanism by which *SlILL6* influences FW potentially through such pathways remains to be determined.

In tomato fruit, the accumulation of metabolites, such as sugars, organic acids, and phenylpropanoids, serves as a critical link connecting genetic information to phenotypic traits including fruit quality and disease resistance [40]. While integrated genomic approaches have proven powerful in identifying trait-associated genes and metabolites, the molecular mechanisms conferring fruit disease resistance in tomato remain incompletely understood. Although several resistance genes, such as *NUCLEOTIDE-BINDING*, *LEUCINE-RICH REPEAT* (*NLR*) genes [41], and *FLS2/FLS3-INTERACTING RECEPTOR-LIKE CYTOPLASMIC KINASE 1* (*FIR1*) [42], have been characterized, these efforts have largely focused on foliar or plant-level immunity, leaving intrinsic fruit resistance mechanisms underexplored. Notably, metabolites, such as chlorogenic acid (CQA) isomers, have been implicated in enhancing disease resistance and fruit quality in solanaceous crops like eggplant [43, 44]. Intriguingly, we detected eight contiguous genes encoding GDSL lipases in one genomic hotspot. Among these eight GDSL lipases, *SlCGT* negatively regulates chlorogenic acid content. As chlorogenic acid inhibits *B. cinerea* growth, this reduction impairs fruit resistance at the turning stage (Fig. 6), consistent with previous studies of fruit preservation and antioxidant protection [45, 46]. While *SlCGT* has been clearly implicated, the functional roles of the other seven GDSL lipases in this cluster remain to be elucidated. Notably, the nonsynonymous mutation in *SlCGT* associated with CQA content aligns with findings in natural tomato populations [47], although its potential co-inheritance with adjacent regulatory elements requires further attention. Thus, our findings not only reinforce the importance of specialized metabolites in pathogen defense but also illustrate how integrated genomic and metabolomic frameworks uncover metabolic pathways underlying complex traits.

In conclusion, our graph-based pangenomics strategy systematically integrated genomics and metabolomics using a RIL population. Genome-wide association analysis (GWAS) confirmed the association of known key loci regulating trichome (*H* gene), vegetative form (*SP*), and FW (*fw2.2*). Furthermore, we identified a novel FW locus, *fw6.4*. Functional validation of the candidate gene *SlILL6* within this locus demonstrated that its overexpression significantly reduces FW, indicating that it acts as a key negative regulator of this trait. We identified 128 differentially accumulated metabolites, whose functions were significantly enriched in the phenylpropanoid biosynthesis pathway. Our mGWAS identified two key enzymatic genes in the pathway, linking *PAL* to phenylalanine metabolism and *F3′H* to kaempferol metabolism. Additionally, we identified and functionally characterized a novel regulator of chlorogenic acid biosynthesis, *SlCGT*. Transgenic analysis demonstrated that *SlCGT* negatively regulates chlorogenic acid accumulation, thereby modulating fruit resistance to pathogenic fungi. These GWAS results were further validated using R/qtl (Fig. S13). By integrating a graph-based pangenome with a multi-omics framework, we systematically decoded the genetic architecture of complex traits in tomato, ranging from morphological development to metabolism. Our work provides valuable genomic resources, analytical tools, and promising candidate genes to support molecular design breeding in tomato.

## Materials and methods

### Population construction and phenotypic investigation

A total of 227 tomato accessions were collected from tomato genetics resource center (TGRC), US department of agriculture (USDA), European Union Solanaceae project (EU-SOL), National Institute for Agricultural Research (INRA) , Institute of Vegetables and Flowers, Chinese Academy of Agricultural Science (IVF-CAAS), and China Agricultural University (CAU). Plant experiments were conducted in the greenhouse at China Agricultural University, Beijing. Plants were grown in a greenhouse at China Agricultural University, Beijing, under controlled conditions (16 h light/8 h dark photoperiod) using a randomized complete block design with three replicates. Phenotypic observations were conducted to record growth type (indeterminate or determinate growth), trichome (thin, dense, or hairless), leaf type (broad, common, thin, or potato), leaf color (green, dark green, or light green), and inflorescence type (single or bifid) for each line. For trait analysis, three representative fruits per plant were randomly selected from three plants per replicate. FW was measured using an electronic balance. Fruit longitudinal and transverse diameters were determined with a digital caliper, and the fruit shape index was calculated. Following transverse sectioning, cross-sectional shape and locule number were recorded. SSC was measured from fruit juice using a handheld refractometer. The mean values across replicates were used for subsequent analyses.

The cultivated tomato (*S. lycopersicum*) line TS-RS1 was hybridized with the wild (*S. pimpinellifolium*) line TS-RS2, generating F_1_ progeny and resulting in the RIL population through the single-seed descent method. Phenotypic evaluation of the 230 F_7_ RILs was conducted in a greenhouse in Beijing using a randomized complete block design with three replicates. For each replicate, three plants per line were sampled, and three representative fruits per plant were collected at the red-ripe stage for trait measurement. Growth type, trichome presence, FW, and SSC were measured following the same procedures as described above.

### Sequencing

To gain a comprehensive understanding of the genetic characteristics of the RIL population, fresh leaf tissues were collected from each line of the RIL population. These leaf samples were then subjected to resequencing using the Illumina NovaSeq 6000 platform. Additionally, a PacBio SMRT library was constructed using high-quality DNA extracted from TS-RS1 and TS-RS2. This library was sequenced on the PacBio Sequel II platform. To investigate the 3D chromatin structure, Hi-C libraries were prepared following the Proximo Hi-C plant protocol. Subsequently, the Hi-C libraries were sequenced on the Illumina NovaSeq platform. RNA was extracted from mixed tissues, including roots, stems, leaves, flowers, and fruits, using the Promega RNG extraction kit (Promega Biotech, Beijing, China). The extracted RNA was sequenced by the Illumina NovaSeq 6000 platform to capture the transcriptome profile.

### Genome assembly

Raw SMRT reads from TS-RS1 and TS-RS2 were corrected and assembled into sequence contigs using CANU [48] with default settings. These contigs were then used in the HERA [49] assembly along with the corrected SMRT reads. To identify overlapping sequences, all contigs and reads were aligned using Minimap2 [50] and Burrows-Wheeler Aligner (BWA) [51] with default parameters. The resulting HERA-assembled super-contigs were combined with BioNano genome maps using IrysView software to create hybrid maps with a minimum length requirement of 150 kb. The contigs derived from this process were further clustered based on Hi-C data using 3D-DNA [52] with default parameters. Lastly, Pilon [53] was employed for additional error correction through three rounds of polishing.

### Genome annotation

The quality of the final genome assembly was evaluated using BUSCO (v2.0) [54], which assesses the presence of Benchmarking Universal Single-Copy Orthologs. To identify interspersed transposable elements (TEs), a combination of *de novo* and homology-based methods was employed. A *de novo* repeat library was generated using LTR finder (v1.07) [55], Repeat Modeler (v2.0.1) [56], and LTR retriever (v2.9.0) [57]. Both the *de novo* library and RepBaseRepeatMaskerEdition-20181026, a widely used repetitive DNA element database, were utilized with RepeatMasker (v4.1.0) [58] to identify TEs.

RNA-seq reads from this study were used to predict protein-coding genes in the repeat-masked genome. To this end, the high-quality RNA-seq reads were aligned to the assembled genome using HISAT2 (v2.1.0) [59] with default parameters. The resulting read alignments were assembled into transcripts using StringTie (v2.1.3b) [60]. The PASA pipeline [61] was then employed to predict complete coding sequences using the assembled transcripts. *Ab initio* gene predictions were performed using software such as BRAKER [62], GeneMark-ET [63], and SNAP [64]. Ultimately, the MAKER pipeline [65] integrated *ab initio* predictions, transcript mapping, and protein homology evidence to generate high-confidence gene models.

### Genome comparisons

To conduct a comprehensive genome-wide comparison between TS-RS1 and TS-RS2 genomes, the MUMmer software package (v3.23) [66] was used. One-to-one alignment blocks were identified using delta-filter. The show-snp tool was employed to detect SNPs and INDELs (<50 bp), focusing on uniquely aligned segments. Moreover, Minimap2 [50] was utilized to obtain information regarding SVs (≥50 bp) between TS-RS1 and TS-RS2. Collinear genes between TS-RS1 and TS-RS2 were identified using MCScan. Specific genes were screened using BLASTp [67] with thresholds of >50% for identity, query coverage, and subject coverage.

### Graph-based pangenome construction and genotyping

The TS-RS1 genome was used as the backbone to construct the graph-based pangenome. This graph integrates small variants (SNPs and INDELs) obtained from the MUMmer (v3.23) [66] and SVs identified by Minimap2 [50] from the genome comparison. These variations were incorporated into the graph using the vg toolkit (v1.35.0) [68] with the ‘vg construct’ command. Key parameters included -r for the reference genome (TS-RS1), -v for the VCF file containing SNPs, INDELs, and SVs. The preliminary graph was simplified with ‘vg prune’, and indexes in XG and GCSA formats were generated using ‘vg index’; the ‘-L’ parameter was applied in both commands.

Genotype matrices for the RIL population were constructed using both the linear and graph-based pangenomes as references. For linear genomes, we identified 230 F_7_ RILs using BWA (v0.7.17) [51] and samtools (v1.10) [69] software with TS-RS1 as the reference genome. High-quality SNPs and INDELs were identified by Genome Analysis Toolkit (GATK, v3.2.2) [70] for variant calling and filtered with thresholds of MAF ≥ 0.05 and missing data rate ≤ 0.2. For the graph-based pangenome, the 230 RILs were sequenced using Illumina paired-end reads, which were subsequently aligned to the graph genome to generate GAM-format alignments. Alignments were filtered to retain only those with mapping quality ≥5 and base quality ≥5. Using default parameters, we then computed a compressed coverage index with ‘vg pack’ and identified snarls with ‘vg snarls’. SNPs, INDELs, and SVs in the RIL population were identified using vg (v1.35.0) giraffe [71], and variants were filtered using the same conditions as for the linear genome.

Genetic linkage mapping was conducted using bin markers derived from the RIL population. Markers with >20% missing data or a MAF below 0.05 were excluded from subsequent analyses. The remaining high-quality bin markers were assigned to 12 linkage groups based on their physical positions on the tomato chromosomes. Marker ordering within each linkage group was refined using the maximum likelihood algorithm implemented in MSTmap [72]. The genetic distances (cM) were calculated using the Kosambi function to account for interference.

### Expression and metabolite profiling

Transcriptome sequencing was conducted on the turning stage fruits using the Agilent 2100 Bioanalyzer platform. The obtained reads were utilized to evaluate gene expression patterns across the entire genome. To quantify the gene expression levels, the reads were aligned to the TS-RS1 genome using HISAT2 (v2.1.0) [59]. The transcripts per million (TPM) value for each gene in different samples was calculated using StringTie (v2.1.3b) [60].

For metabolite profiling, fruit samples were collected from the F_7_ RIL population grown in the greenhouse in Beijing, as described above. Three biological replicates were performed for each line, with each replicate consisting of a pooled sample of three or more fruits harvested at the turning stage. Fresh fruits were immediately frozen in liquid nitrogen and subjected to freeze-drying prior to metabolite extraction. We analyzed tomato fruit metabolites using the UltiMate 3000 UHPLC system coupled to a Q-Exactive Orbitrap mass spectrometer (Thermo Fisher Scientific Ltd, CA, USA), under both positive and negative modes. Extracts were separated on an ACQUITY UPLC™ BEH C18 column (Waters Ltd, MA, USA) (150 × 2.1 mm, 2.6 μm). Solvents for the mobile phase were 0.05% formic acid in acetonitrile (A) and 0.1% formic acid in water (B). A 25-min gradient was used to separate the samples. Data with mass ranges of *m*/*z* 80–1200 were acquired separately in both positive ion mode and negative ion mode, with data-dependent MS/MS acquisition. TraceFinder analysis software (version 4.1) was used to analyze the MS and MS/MS data acquired. Metabolites from the MS and MS/MS data were annotated based on commercial databases and in-house databases *via* high-resolution MS-associated methods. Then, MetaboAnalyst 4.0 was used for downstream analysis.

### QTL (quantitative trait locus) mapping

GWAS was performed using variant matrices constructed from both the TS-RS1 linear genome and the MiniTG1.0 graph-based pangenome as genotypes, with MLM analysis conducted *via* EMMAX software [21]. A Balding–Nichols kinship matrix was constructed from the filtered variants using default parameters to account for relatedness among individuals. The principal components were included as fixed effects. A significance level of 0.05 was applied for single tests. To correct for multiple testing, the genome-wide significance threshold was calculated using GEC software (Genetic type I Error Calculator v0.2; http://grass.cgs.hku.hk/gec/register.php), which estimates the effective number of independent variants. The resulting threshold was *P* < 2.74 × 10^−6^. GWAS results were visualized using a Manhattan plot generated using ‘CMplot’ (https://cran.r-project.org/web/packages/CMplot/). For the top signals, the corresponding bin marker intervals were retrieved from the TS-RS1 reference genome. All annotated protein-coding genes located within these intervals were extracted, and candidate genes were prioritized by integrating differential expression, functional annotations, and relevant literature mining.

QTL mapping was performed using the R package qtl (v1.50) [73]. The genetic map and phenotypic data from the 230 F_7_ RILs were imported using read.cross(). Composite interval mapping was conducted with the cim() function.

### Reverse transcription quantitative polymerase chain reaction analysis

Total RNA was extracted from the pericarp of fruits at different developmental stages using the Quick RNA Isolation Kit (Huayueyang Biotechnology Company). The RNA was then reverse transcribed using the PrimeScript™ RT reagent kit (TaKaRa). The relative expression of target genes was quantified using the ABI QuantStudio™ 6 Flex (Applied Biosystems, CA, USA). The reverse transcription quantitative polymerase chain reaction (qRT-PCR) was carried out using a TB Green® Premix EX Taq™ kit (TaKaRa). The internal control used for the qRT-PCR was *SlEXP* (*Solyc07g025390*). Relative expression was calculated using the 2^−ΔΔCT^ method. All primers used in the experiment are listed in Table S8. The *P*-value determined by two-sided Student’s *t*-tests < 0.05 (*P* < 0.05) was considered statistically significant.

### Generation of *SlCGT* and *SlILL6* mutants

CRISPR/Cas9-mediated gene editing was performed using cultivated tomato (*S. lycopersicum*). To disrupt *SlCGT*, two sgRNAs were designed and their activity was assessed through *in vitro* DNA-cleavage assays using Cas9–gRNA ribonucleoprotein complexes and the CYC-B DNA template. The transformation was carried out using a binary vector containing a polycistronic sgRNA construct and a Cas9 expression cassette. Homozygous mutants of both genotypes with 1 and 5 bp deletions, free of T-DNA, were obtained within the first two generations after plant transformation.

To obtain overexpressed lines, the *SlCGT* coding sequence was cloned into the pEASY cloning vector. Finally, the electroporation method was used to transfer the plasmid into *Agrobacterium tumefaciens* and then transformed into cultivated tomato (*S. lycopersicum*) cv. Ailsa Craig. The *SlILL6* overexpression vector was constructed using the In-fusion method, and the CDS was cloned into the pEGHPB cloning vector. The vector was constructed using the Seamless Assembly Cloning Kit and transferred into *A. tumefaciens* strain GV3101 using the chemical transformation method and then transformed into cultivated tomato (*S. lycopersicum*) cv. M82 (Table S8).

### *Botrytis cinerea* inoculation assays

A 5 μl of resuspended spore solution of *B. cinerea* gray mold (5 × 10^5^) was spotted in the center of half-strength PDB medium containing different concentrations of chlorogenic acid (0, 100, 200, and 300 μg/ml), forming a round droplet. The surface of the medium was blown dry, sealed, and then placed in an inverted incubator at 22°C under a 16/8 h light/dark photoperiod. When the 0 μg/ml medium was fully covered with mycelium, the size of mycelium diameter on the plates containing different concentrations of chlorogenic acid was recorded. To assess the role of *SlCGT* in tomato disease resistance, 5 μl of *B. cinerea* was transferred onto preinjured tomato fruits in a growth chamber at 22°C with a 16/8 h light/dark photoperiod. Lesion diameters were measured with a ruler after 48 h. Three independent biological replicates were conducted.

## Supplementary Material

Web_Material_uhag147

## Data Availability

All genomes, raw sequencing data for genome assembly and annotation have been deposited in the BIG Data Center (https://bigd.big.ac.cn) under Accession Number PRJCA032521 and in the National Center of Biotechnology Information (https://www.ncbi.nlm.nih.gov) under Accession Number PRJNA1190221. Metabolomics data have been deposited in the Open Archive for Miscellaneous Data (OMIX) database at the BIG Data Center under Accession Number OMIX008028. Custom scripts and codes used in this study are provided at GitHub (https://github.com/Lintao1987/MiniTG). Software and tools used are described in the Methods.
