# Supplementary material for: Graph-based pangenomics reveals the genetic basis of agronomic traits in tomato fruits
Source: Hortic Res. 2026 Apr 16;13(9):uhag147. doi: 10.1093/hr/uhag147 (PMC13503963; doi:10.1093/hr/uhag147)
Supplement: Supplementary file 1 [file web_material_uhag147.zip › Supplementary Figures 1-13 v2.docx]

**Supplementary Figures 1-13**

**Figure S1. Layout of the minigraph-based genome (MiniTG1.0) study.** **A**. Parental selection and RIL population construction. **B**. Construction of graphical genome and preparation of multi-omics data. **C**. Variation matrix and mGWAS analysis. **D**. Molecular identification, molecular breeding, and metabolic engineering.

**Figure S2. Phylogenetic tree of 227 tomato accessions comprising two phylogenetic groups (*S. pimpinellifolium* (PIM) and *S. lycopersicum* (BIG)).**

**Figure S3. Comparison of variation between parents and RIL population. A**. Variation number of parents and RIL population. **B**. Comparison of MAF between RIL-specific SVs and all SVs (***, *P*-value < 0.001). **C**. IGV visualization of representative RIL-specific SVs (Chr1:44235938, Chr8:44235938, and Chr12:65062035).

**Figure S4. Construction of genetic map. A**. Graphical representation of the re-sequencing-based mapping results of 230 RIL accessions. **B**. Collinearity analysis between genetic map and genome.

**Figure S5. Manhattan plots of the trichome and vegetative form in the TS-RS1 and MiniTG1.0. A**. Manhattan plot of trichome phenotype in the GWAS of the TS-RS1 linear genome. **B**. Manhattan plot of trichome phenotype in the GWAS of the MiniTG1.0. Top, the phenotype of trichome-bearing accession TS-RS1 and trichomeless accession TS-RS2. Scale bars: 1 cm. **C**. Identification of a deletion of the *H* gene. **D**. Manhattan plot of vegetative form phenotype in the GWAS of the TS-RS1 linear genome. **E**. Manhattan plot of vegetative form phenotype in the GWAS of the MiniTG1.0. **F**. Identification of *SP* gene.

**Figure S6. The relative expression levels of *SlILL6* in the different tomato fruit developmental stages.** Data are presented as mean ± s.e.m. (n = 3). Statistical significance was determined by two-sided Student’s *t*-tests (*, *P*-value < 0.05; **, *P*-value < 0.01; n.s., not significant).

**Figure S7. Comparison of fruit transverse diameter (A), longitudinal diameter (B), and shape index (C) between wild-type and overexpression lines.** Data are presented as mean ± s.e.m. (n = 16). Statistical significance was determined by two-sided Student’s *t*-tests (***, *P*-value < 0.001; n.s., not significant).

**Figure S8. Coefficient of variation of metabolites in the RIL population.**

**Figure S9. Dissection of the *SlPAL* gene regulating Primary metabolism of phenylpropanoids.** **A**. Correlation and Gaussian distribution of *SlF3'H* expression, and the contents of phenylalanine, cinnamic acid, and *p*-coumaric acid. **B**. Manhattan plots of GWAS for phenylalanine, cinnamic acid, and *p*-coumaric acid. **C-E**. Allele distribution in varieties containing R or A allele combinations associated with phenylalanine (**C**), cinnamic acid (**D**), and *p*-coumaric acid (**E**). R is the wild TS-RS2 allele and A is the cultivated TS-RS1 allele. The statistical significance was determined by two-sided Student’s *t*-tests (*, *P*-value < 0.05; ***, *P*-value < 0.001).

**Figure S10. Dissection of the *SlF3'H* gene regulating kaempferol content.** **A**. Correlation and Gaussian distribution of the contents of kaempferol, kaempferol 3-O-glucoside, kaempferol 3-glucorhamnoside, and camelliaside B. **B**. Manhattan plots of GWAS for kaempferol and its glycosides. **C**-**F**. Allele distribution in varieties containing R or A allele combinations associated with kaempferol (**C**), kaempferol 3-O-glucoside (**D**), kaempferol 3-glucorhamnoside (**E**), and camelliaside B (**F**). R is the wild TS-RS2 allele and A is the cultivated TS-RS1 allele. The statistical significance was determined by two-sided Student’s *t*-tests (***, *P*-value < 0.001).

**Figure S11. Fruit soluble solids content of *SlCGT* transgenic lines.** Data are presented as mean ± s.e.m. (n = 3). Statistical significance was determined by two-sided Student’s *t*-tests (n.s., not significant).

**Figure S12. The inhibitory effect of chlorogenic acid on the growth of *B. cinerea*. A**. Growth of *B. cinerea* in medium containing 0, 100, 200, and 300 μg/mL of CGA (n=3). Scale bars: 1 cm. **B**. Effect of CGA immersion on lesion diameter in turning-stage fruits of wild lines inoculated with *B. cinerea* (n=9). Data are presented as mean ± s.e.m. Statistical significance was determined by two-sided Student’s *t*-tests.

**Figure S13. QTL mapping across the entire RIL population for trichome (A), vegetative form (B), fruit weight (C), kaempferol and its three glycosides (D), three phenylpropanoid-related primary metabolites (E), and chlorogenic acid (F).**

**Figure S1. Layout of the minigraph-based genome (MiniTG1.0) study.** **A**. Parental selection and RIL population construction. **B**. Construction of graphical genome and preparation of multi-omics data. **C**. Variation matrix and mGWAS analysis. **D**. Molecular identification, molecular breeding, and metabolic engineering.

**Figure S2. Phylogenetic tree of 227 tomato accessions comprising two phylogenetic groups (*S. pimpinellifolium* (PIM) and *S. lycopersicum* (BIG)).**

**Figure S3. Comparison of variation between parents and RIL population. A**. Variation number of parents and RIL population. **B**. Comparison of MAF between RIL-specific SVs and all SVs (***, *P*-value < 0.001). **C**. IGV visualization of representative RIL-specific SVs (Chr1:44235938, Chr8:44235938, and Chr12:65062035).

**Figure S4. Construction of genetic map. A**. Graphical representation of the re-sequencing-based mapping results of 230 RIL accessions. **B**. Collinearity analysis between genetic map and genome.

**Figure S5. Manhattan plots of the trichome and vegetative form in the TS-RS1 and MiniTG1.0. A**. Manhattan plot of trichome phenotype in the GWAS of the TS-RS1 linear genome. **B**. Manhattan plot of trichome phenotype in the GWAS of the MiniTG1.0. Top, the phenotype of trichome-bearing accession TS-RS1 and trichomeless accession TS-RS2. Scale bars: 1 cm. **C**. Identification of a deletion of the *H* gene. **D**. Manhattan plot of vegetative form phenotype in the GWAS of the TS-RS1 linear genome. **E**. Manhattan plot of vegetative form phenotype in the GWAS of the MiniTG1.0. **F**. Identification of *SP* gene.

**Figure S6. The relative expression levels of *SlILL6* in the different tomato fruit developmental stages.** Data are presented as mean ± s.e.m. (n = 3). Statistical significance was determined by two-sided Student’s *t*-tests (*, *P*-value < 0.05; **, *P*-value < 0.01; n.s., not significant).

**Figure S7. Comparison of fruit transverse diameter (A), longitudinal diameter (B), and shape index (C) between wild-type and overexpression lines.** Data are presented as mean ± s.e.m. (n = 16). Statistical significance was determined by two-sided Student’s *t*-tests (***, *P*-value < 0.001; n.s., not significant).

**Figure S8. Coefficient of variation of metabolites in the RIL population.**

**Figure S9. Dissection of the *SlPAL* gene regulating Primary metabolism of phenylpropanoids.** **A**. Correlation and Gaussian distribution of *SlPAL* expression, and the contents of phenylalanine, cinnamic acid, and *p*-coumaric acid. **B**. Manhattan plots of GWAS for phenylalanine, cinnamic acid, and *p*-coumaric acid. **C-E**. Allele distribution in varieties containing R or A allele combinations associated with phenylalanine (**C**), cinnamic acid (**D**), and *p*-coumaric acid (**E**). R is the wild TS-RS2 allele and A is the cultivated TS-RS1 allele. The statistical significance was determined by two-sided Student’s *t*-tests (*, *P*-value < 0.05; ***, *P*-value < 0.001).

**Figure S10. Dissection of the *SlF3'H* gene regulating kaempferol content.** **A**. Correlation and Gaussian distribution of the contents of kaempferol, kaempferol 3-O-glucoside, kaempferol 3-glucorhamnoside, and camelliaside B. **B**. Manhattan plots of GWAS for kaempferol and its glycosides. **C**-**F**. Allele distribution in varieties containing R or A allele combinations associated with kaempferol (**C**), kaempferol 3-O-glucoside (**D**), kaempferol 3-glucorhamnoside (**E**), and camelliaside B (**F**). R is the wild TS-RS2 allele and A is the cultivated TS-RS1 allele. The statistical significance was determined by two-sided Student’s *t*-tests (***, *P*-value < 0.001).

**Figure S11. Fruit soluble solids content of *SlCGT* transgenic lines.** Data are presented as mean ± s.e.m. (n = 3). Statistical significance was determined by two-sided Student’s *t*-tests (n.s., not significant).

**Figure S12. The inhibitory effect of chlorogenic acid on the growth of *B. cinerea*.** **A**. Growth of *B. cinerea* in medium containing 0, 100, 200, and 300 μg/mL of CGA (n=3). Scale bars: 1 cm. **B**. Effect of CGA immersion on lesion diameter in turning-stage fruits of wild lines inoculated with *B. cinerea* (n=9). Data are presented as mean ± s.e.m. Statistical significance was determined by two-sided Student’s *t*-tests.

**Figure S13. QTL mapping across the entire RIL population for trichome (A), vegetative form (B), fruit weight (C), kaempferol and its three glycosides (D), three phenylpropanoid-related primary metabolites (E), and chlorogenic acid (F).**
